# An Epidemiological Survey to Investigate the Prevalence of Cystic Echinococcosis in Slaughtered Bovine Hosts in Punjab, Pakistan

**DOI:** 10.3390/vetsci10010040

**Published:** 2023-01-05

**Authors:** Sadia Saleem, Haroon Ahmed, Kaleem Imdad, Jing Zhang, Jianping Cao

**Affiliations:** 1 Department of Biosciences, COMSATS University Islamabad, Islamabad 45550, Pakistan; 2 Key Laboratory of Parasite and Vector Biology, National Health Commission of People’s Republic of China, Shanghai 200025, China; 3 National Institute of Parasitic Diseases, Chinese Center for Disease Control and Prevention, Chinese Center for Tropical Diseases Research, Shanghai 200025, China; 4 WHO Collaborating Centre for Tropical Diseases, Shanghai 200025, China; 5 The School of Global Health, Chinese Center for Tropical Diseases Research, Shanghai Jiao Tong University School of Medicine, Shanghai 200025, China

**Keywords:** cystic echinococcosis, hydatid disease, Punjab, cattle, dog, Pakistan

## Abstract

**Simple Summary:**

Cystic echinococcosis (CE) is a zoonotic disease of human and animals having worldwide geographical distribution resulting in major economic losses. It is most common in underdeveloped and herding communities where people survive on animal husbandry and agricultural activities. The prevalence of CE in livestock and its risk factors are widely underreported. Therefore, this study aimed to evaluate the epidemiological characteristics and prevalence of disease in cattle in Punjab Pakistan. The study was conducted in the Punjab province of Pakistan. The data were collected from slaughterhouses from September 2021 to February 2022. Antemortem and postmortem examination and cyst characterization was conducted. A questionnaire was designed to collect epidemiological, demographic, and primary health data. A total of 8877 animals were examined, and the prevalence was 6.22% in all the examined animals, with a higher prevalence in cattle. There is a slight difference in the prevalence among males and females. Animals were examined at three different slaughterhouses in the Rawalpindi city originating from 23 districts of Punjab, Khyber PakhtunKhwa (KP) and Azad Jammu and Kashmir (AJK). The highest prevalence was recorded in animals from the Haripur district of KP. The majority of animals studied were older than 3 years, also most infected animals studied were from herds. Predominantly, cysts were observed in the lungs and liver. These findings may be useful in estimating the eco-epidemiology of CE and improving surveillance and prevention programs in Pakistan.

**Abstract:**

Cystic echinococcosis (CE) is a neglected zoonotic disease of worldwide geographical distribution. CE is most common in underdeveloped and herding communities where people survive on animal husbandry and agricultural activities. The prevalence of CE in livestock and its risk factors are widely underreported, because of inefficient surveillance systems. The aim of this study was to evaluate the epidemiological characteristics and prevalence of CE in cattle in Punjab, Pakistan. Data were collected from slaughterhouses from September 2021 to February 2022. Ante- and postmortem examination and cyst characterization were performed. Epidemiological, demographic, and one health data were collected. A total of 8877 animals (8096 buffalo, 781 cattle) were examined, and the prevalence of CE was 6.22% (*n* = 552) in all animals, with a higher prevalence in cattle (15.20% vs. buffalo 5.83%). Prevalence was not significantly different in males and females. Of the 23 districts studied, the highest prevalence was in the Haripur district of KP (20.85%). The majority of animals studied were older than 3 years. Most cysts were found in animals older than 5 years. Lungs and liver were the predominant sites for the presence of cysts (65.58% and 31.34%, respectively. Of the collected cysts, 29.71% were fertile. The findings may be useful in estimating the eco-epidemiology of CE and improving surveillance and prevention programs in Pakistan.

## 1. Introduction

Cystic echinococcosis (CE), commonly called hydatid disease, is a common zoonotic infection caused by the larval stage of a taeniid tapeworm, *Echinococcus granulosus* species complex [1]. As per molecular and epidemiological studies, five species are responsible for most infections: *E. granulosus sensu stricto* (G1–G3), *E. equinus* (G4), *E. ortleppi* (G5), *E. canadensis* (G6/G7, G8, G10), and *E. felidis* [2]. Among these, G1 is responsible for 88.4% of human CE cases [3].

CE is designated as a neglected zoonotic disease by the World Health Organization and has worldwide geographical distribution. It is particularly prevalent in Africa, Asia, Australia, Europe, central and southern regions of the United States of America, and specifically the United Kingdom, the Mediterranean region, Iran, Kuwait, Saudi Arabia, Iraq, Syria, Jordan, and Pakistan, resulting in major damage to livestock [4,5,6]. CE is most common in underdeveloped and herding communities where people survive on animal husbandry and agricultural activities [1] because the distribution and prevalence of CE in any region depend upon factors such as the presence of stray dogs in the population, inadequate anthelminthic treatment of dogs and other animals, production of livestock on a large scale, poor standards of hygiene and cleanliness, the availability of offal of slaughtered animals to dogs, inappropriate or absent inspection of slaughterhouses, open slaughtering practices with no veterinary supervision, and lack of disposal of slaughter waste products [7,8,9].

The CE cycle is maintained by domestic animals such as cattle, swine (intermediate host), and dogs (definitive host). The disease is transmitted through the fecal-oral route. Herbivores become infected by ingesting tapeworm eggs present in pasture grasslands that later develop and form cysts ranging from the size of a pea to that of a medium-sized football. These develop in the vital organs, mostly the lungs and liver, although also, rarely, in kidneys, spleen, bone, brain, and heart. Dogs acquire the infection by eating the infected organs of slaughtered or dead animals. Humans are accidental hosts and become infected by ingesting *E. granulosus* eggs through food, water, or soil contaminated with eggs passed into the feces of the definitive host. Cysts may be asymptomatic and remain unnoticed for a long time. Symptoms are variable, depending on the location, size, and nature of the cyst [10,11].

CE causes serious damage around the globe to the general economy and to the agriculture and health sectors particularly. Annually, 1.2 million people become infected and the death rate reaches 2.2%; in addition, it results in 3.6 million disability-adjusted life years (DALY). Globally, USD 3 billion is spent on managing CE per annum [12,13]. In Pakistan, CE costs 26.5 million rupees annually; additional economic losses per 100 sheep-goats and buffalo cattle were estimated to be USD 276.20 and USD 165.72, respectively [14].

Being an agricultural country, a major portion of Pakistan’s economy depends on livestock production; however, the output is considerably reduced owing to the prevalence of many parasitic infections such as CE, the infection rates of which range from 2.44% to 35% in Pakistan [15,16,17,18]. To implement control measures the reported prevalence of CE in livestock and knowledge of its risk factors are required [6]. These are currently widely underestimated, because of inefficient surveillance systems based on reports from slaughterhouses. Therefore, this study aimed to evaluate the epidemiological features and prevalence of CE in bovine hosts in Punjab, Pakistan.

## 2. Materials and Methods

### 2.1. Study Region

This study was conducted in the Punjab province of Pakistan. Punjab (31° N, 72° E) is the second largest and most densely populated province of Pakistan. It is bordered by India in the east and the India-administered territories of Jammu and Kashmir in the northeast. It comprises the fertile alluvial plains of the river Indus and its tributaries. It also includes mountainous ranges along with the desert region. Administratively, Punjab consists of 10 divisions comprising 36 districts, with Lahore as its capital. Most areas in Punjab experience extreme weather, with foggy winters often accompanied by rain.

### 2.2. Study Duration

The data in this study were collected between September 2021 and February 2022. Data and samples were collected from three major urban slaughterhouses in the Rawalpindi district where cattle from across Punjab are brought for slaughter.

### 2.3. Sample Collection and Storage

During antemortem examination, each animal was checked for any physical abnormalities. Through visual inspection during postmortem examinations, cattle were examined for the presence of cyst: cysts, organ palpation was performed in case suspicious anatomical structures were seen, and all cyst-containing organs (liver, lungs, spleen, muscles, kidney, and heart) were collected into sterile zip-locked polythene bags. Samples were labeled with specific IDs and were stored temporarily at 4 °C.

### 2.4. Cyst Characteristics

Organs were later incised and the cysts were isolated for further characterization. For proper examination of hydatid cysts, data on the following parameters were noted: organ specificity, type of cysts (sterile, fertile, calcified, or underdeveloped), and the number of cysts. The size of the cyst after dissection (using a ruler) and the appearance/color of the cyst after isolation was also recorded.

For cyst characterization, cysts were dissected using sterile scalpel blades and collected into separate sterile containers. Fluid present in the cyst was collected in 1.5-mL Eppendorf tubes and the fertility of the cyst was measured on the basis of the existence of protoscoleces as brood capsules or in the cyst fluid [19]. To test viability, equal volumes of cyst fluid and eosin solution were mixed and amoeboid or peristaltic movements were observed in fluid containing protoscoleces using microscopy. Characterization of sterile cysts was carried out on the basis of their smooth inner lining, with turbid and comparatively less fluid. Calcified cysts were nodular with calcified deposition on their inner walls [20,21]. Cysts ranging in size between 1 mm and 2 mm were characterized as underdeveloped; protoscoleces were not found in these cysts, which had a firm texture with a minute amount of fluid.

### 2.5. Collection of Demographic, Epidemiological, and One Health Data

Two different questionnaires were designed to collect epidemiological, demographic, and one health data. The first questionnaire was designed for animal owners who had accompanied their livestock to slaughterhouses. If necessary, owners were interviewed in their local language (usually Punjabi or Urdu). Data collected included animal age, color, and gender; number of animals in the herd; place of origin of the animal. A second questionnaire (one health) was used to interview butchers and abattoir workers at the three slaughterhouses. Data regarding the number of dogs in the vicinity of the slaughterhouse, disposal of offal, veterinary supervision of slaughtering, access of stray dogs to infected organs, and anthelminthic treatment of stray dogs was recorded [22].

### 2.6. Statistical Analysis

Descriptive statistics and a Chi-square test were used to analyze the data using SPSS 16 software, with the significance level set at *p* < 0.05.

## 3. Results

A total of 8877 animals (8096 buffalo, 781 cattle) were examined. The prevalence of CE was 6.22% (*n* = 552) in all animals examined, with prevalence in cattle at 15.20%, which was significantly higher than that of buffalo at 5.83% (*p* = 0.00001; Table 1).

**Figure 1:**
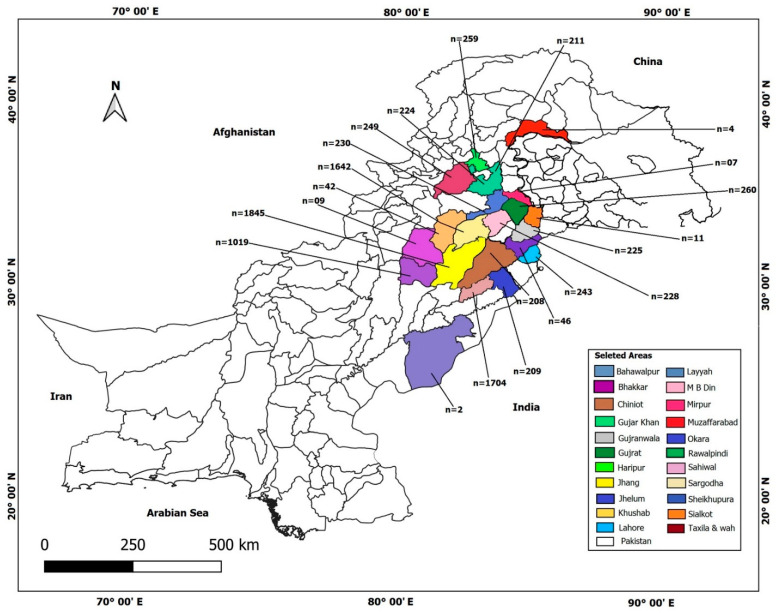
Map of Pakistan depicting distribution of animals examined (n is number of animals examined per district).

With respect to sex, no significant differences in prevalence were observed, at 6.25% for male and 5.90% for female animals (*p* = 0.7263); however, in both host species, infection was more prevalent in males than in females. Most animals examined were from the Punjab province (*n* = 8612); however, some animals were recorded as coming from the Khyber PakhtunKhwa (KP) province (*n* = 256) and the state of Azad Jammu and Kashmir (AJK) (*n* = 9) and were brought to Punjab to slaughter. According to the responses of animal owners, animals from 23 districts were examined at the slaughterhouses (Figure 1). The highest prevalence of CE was calculated for animals from the Haripur district of KP (20.85%) followed by Taxila in Punjab (16.32%), Mirpur of AJK (14.29%), and Gujrat (13.46%), Lahore (13.17%), Rawalpindi (10.43%), Wah Cantt (10.0%), Sheikhupura (8.70%), Sargodha (7.31%), and Okara (4.31%) in Punjab, respectively (*p* = 0.00; Table 1).

The majority of animals were older than 3 years, and only a few animals (less than 10) were slaughtered at younger ages. The highest infection rate was recorded in animals aged 9 years (11.76%), followed by 7 years (9.67%), 8 years (7.52%), and 6 years (6.06%; Table 1).

Data on livestock farming were collected: 41.13% of slaughtered animals were from herds of 6–15 animals followed by 16.71% from herds of 16–50 animals, 2.50% from herds of more than 100 animals, and 2.46% from herds of 50–100 animals. Only 0.06% of total animals were from very small herds of 2–5 animals and a majority of animals (37.15%) of animals had been kept as single animals. In summary, single-kept animals were far less often infected than animals living in herds, the highest infection rate was seen in herds of 16–50 animals (Table 1).

The monthly prevalence of hydatid cysts in slaughtered animals was also recorded. A maximum prevalence of 7.66% was recorded in October 2021, followed by 6.91% in January 2022, 6.55% in November 2021, 6.42% in September 2021, and 5.52% in December 2021. The lowest prevalence, 4.97%, was recorded in February 2022 (Table 2).

A total of 552 animals were found to have cysts (6.22%) out of 8877 animals examined. Of these animals, 65.58% had a single infection in the lung followed by 31.34% in the liver, 1.09% in the thorax, 0.54% in the heart, 0.36% in the pancreas, and 0.18% each in the kidneys and skeletal muscles and, 0.72% had a double infection in both lungs and liver. The color of the cyst recorded after isolation ranged from transparent (2.72%), white (37.68%), yellow (2.36%), pale yellow (53.99%), pinkish yellow (0.36%), reddish (0.36%), green (2.72%), and gray (1.81%), to black (0.18%; Table 3).

**Figure 2:**
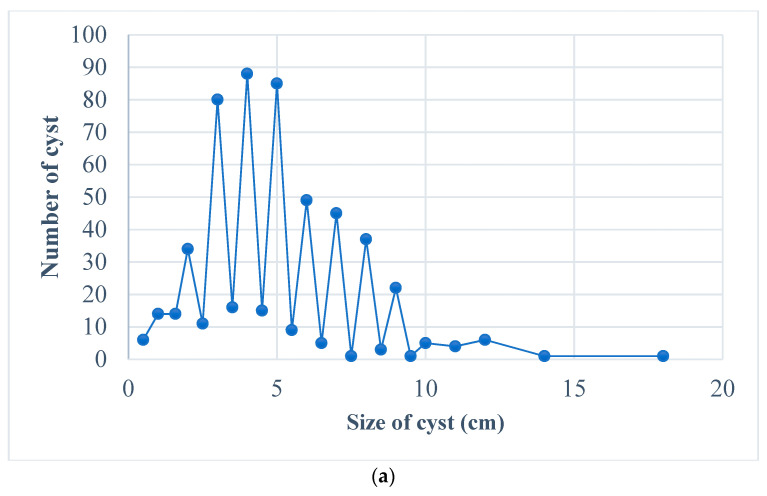

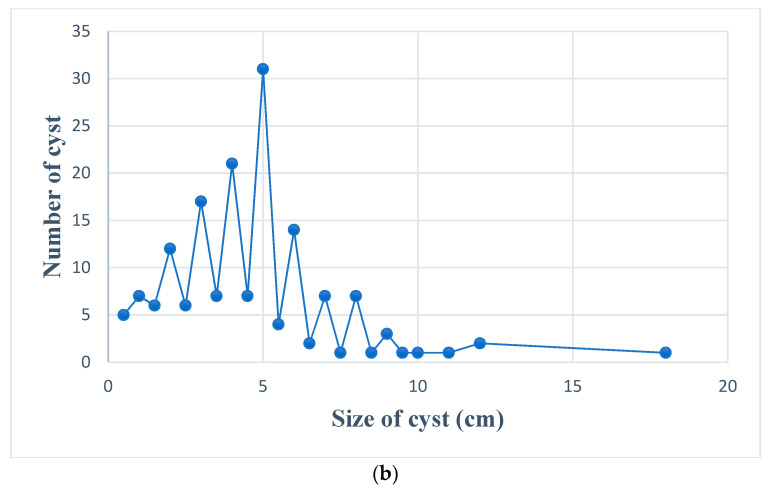
Variation in cyst size, (**a**). all cyst, (**b**). fertile cyst.

Of all cysts examined, 45 were calcified and 507 were non-calcified. In terms of fertility evaluation, 29.71% (*n* = 164) of cysts were found to be fertile whereas 70.29% (*n* = 388) were unfertile. As fertile cysts are most important for the epidemiology of CE, these cysts were characterized separately and are represented in Table 3. The rate of fertility in cattle is greater than that in buffalo. The size of the cyst in centimeters was also measured, and cysts ranged in size from as small as 0.18 cm up to 15.94 cm, 46.2% of infected animals more than 6 years of age have cyst sizes > 5 cm. A comparison in size of all cysts and fertile cysts is shown in Figure 2.

As dogs are one of the major sources of human CE, the slaughterhouses were inspected for possible risk factors for parasite transmission. According to the data from the second questionnaire, there is a considerable risk of local dogs becoming infected due to the lack of hygiene, education, and control in the three slaughterhouses of Rawalpindi (Table 4).

## 4. Discussion

CE poses a serious health and economic threat. Despite its high prevalence in many societies, it remains a neglected zoonotic disease, with few surveillance studies to provide an accurate picture of its prevalence, endemic strains, and associated risk factors [23,24]. CE is most prevalent in low-income countries. Accordingly, a high prevalence of CE infection has been reported in Pakistan in many studies, owing to factors such as low socioeconomic development leading to poor health and living conditions for the majority of people, poor hygiene practices, lack of appropriate checks and balances in slaughtering, poor cleanliness of shops, poor management of stray dogs, and the unavailability of clean drinking water to many [25,26,27,28,29,30,31,32,33]. The first study investigating the prevalence of CE in animals in Pakistan was conducted in 1968 in Lahore, in which a CE prevalence of 35% was recorded for buffalo and 27% for cattle [30]. In the present study, CE prevalence was recorded as 6.22% (*n* = 552) in all animals examined, with prevalence in cattle at 15.20% and buffalo at 5.83%. Similar prevalence in cattle has been reported in studies conducted in different districts of the KP province [18,34].

With respect to the sex of animals, we found infection more prevalent in males than females, this was also consistent with the presence of fertile cysts. These findings are contrary to previous findings, where females were affected more, by Lemma et al. [35] and Banda et al. [36].

In this study, animals from 23 districts in the province of Punjab, KP, and the state of AJK were examined. The major proportion of animals was from central and southern Punjab areas such as Sargodha, Gujrat, Jhang, and Layyah, which are the agricultural centers of the province where cattle rearing is common. The differences in prevalence in districts are mainly due to geographical distribution, versatile social and cultural activities, and differences in husbandry techniques, hygiene practices, and the rearing of dogs [34,37]. High prevalence in Haripur and Gujrat can be due to more rural populations in these districts, where old husbandry techniques are most common, leading to the spread of infection [38,39].

The majority of animals came from herds. It is a regular practice of farmers to keep animals in herds along with herding dogs, and this could be a major factor in the transmission of CE, as animals in herds are exposed to similar environments and grazing pastures. In this study, CE infection was revealed to be more prevalent in animals older than 5 years, and this is similar to results reported in studies on bovines in Hyderabad, Pakistan, Morocco, and Ethiopia [15,40,41]. This could be explained by longer exposure to infection and easier recognition of cysts, owing to the larger size of cysts in older animals [34]. During the period of this study highest number of positive animals was recorded in October, whereas the lowest was recorded in February. Monthly prevalence is not significantly associated with cystic echinococcosis, as it is a chronic infection and cysts take a long time to develop; it could only be of importance that survival of hydatid cyst in discarded organs varies with temperature, it could survive for many days in cold weather and poses a threat for infection in stray dogs and remains viable for only a few hours in hot weather [42].

In the 552 cyst-carrying animals, for cyst development, the lungs were infected twice as much compared to the liver (65.58% vs. 31.35%) Heart, spleen, and kidneys followed, similar to findings reported in studies from the European Mediterranean area, Uganda, and Ethiopia [43,44,45]. Moreover, of the cysts collected, 29.71% were fertile, which is similar to findings in KP, Pakistan [34]. The different developmental stages of the cysts detected (fertile, sterile, calcified) may, among other reasons, be due to strain differences of *E. granulosus* [45]. The elevated infection rate seen in cattle might result from specific local conditions at the place of their origin or a species-specific immune reaction towards the parasite. It is also of interest that more fertile cysts were detected in infected cattle than in buffaloes (45% vs. 27.1%)—hence infected cattle organs could pose a more serious threat for canine infection than organs from buffaloes.

Dogs were found around slaughterhouses throughout the study period. Although it was claimed that offal was being sold, it was observed that dogs accessed some of the meat remains, and other practices could be contributory factors for the spread of disease, as discussed in previous studies [7,46,47,48].

## 5. Conclusions

To the best of the knowledge of the authors, this study is the first to estimate the prevalence of bovine CE in Punjab, Pakistan, and the study covered more than 20 districts. Further studies on all animal hosts along with the findings of this study can be used in the future for estimating the eco-epidemiology of CE and to improve surveillance and prevention programs in Pakistan.

## Figures and Tables

**Table 1:** Host factors and the related infection rate of cattle and buffaloes seen at the slaughterhouses in Rawalpindi district.

No. of Animals Examined	Cattle		Buffalo		Test Statistic *(χ2)*
Epidemiological Factors	Examined	Positive Percentage	Examined	Positive Percentage	
District					
Bahawalpur	0	0	2	0.00	*χ2* = 370.99 df = 22 *p* = 0.00
Bhakkar	2	50.00	7	14.29	
Chiniot	21	9.52	187	5.35	
Gujar Khan	15	20.00	209	2.87	
Gujranwala	14	21.43	211	5.21	
Gujrat	27	22.22	233	12.45	
Haripur	23	39.13	236	19.07	
Jhang	172	5.23	1673	2.57	
Jhelum	17	11.76	213	5.63	
Khushab	3	0.00	39	7.69	
Lahore	21	33.33	222	11.26	
Layyah	90	8.89	929	4.31	
M B Din	15	0.00	213	1.88	
Mirpur	1	0.00	6	16.67	
Muzafarabad	0	0	4	0.00	
Okara	14	14.29	195	8.72	
Rawalpindi	33	12.12	178	10.11	
Sahiwal	142	5.63	1562	3.71	
Sargodha	143	9.79	1499	7.07	
Sheikupura	9	0.00	37	10.81	
Sialkot	0	0	11	9.09	
Taxila	19	10.53	220	16.82	
Wah cantt	0	0	10	10.00	
Herd					
Single	287	0.35	3011	0.10	*χ2* = 435.4869 df = 5 *p* = 0.00
2–5 animals	4	0.00	1	100.00	
6–15 animals	309	14.24	3342	7.24	
16–50 animals	134	19.40	1349	14.83	
50–100 animals	22	22.73	196	6.63	
>100	25	16.00	197	6.60	
Age					
3–5 years	207	1.93	1952	1.18	*χ2* = 193.34 df = 5 *p* = 0.00
6 years	106	8.49	1016	5.81	
7 years	105	20.00	1033	8.62	
8 years	180	12.78	2094	7.07	
9 years	95	17.89	1044	11.21	
≥10 years	88	6.82	957	3.76	
Gender					
Male	369	13.82	7241	5.87	*χ2* = 0.1225 df = 1 *p* = 0.7263
Female	412	7.04	855	5.50	

**Table 2:** Monthly prevalence of hydatid cysts in slaughtered animal species.

Cattle				Buffalo			
Month	Examined	Positive	Prevalence	Examined	Positive	Prevalence	Test Statistics (*χ2*)
Sep	141	13	9.22	1137	69	6.07	*χ2*= 10.90 df = 5 *p* = 0.0533
Oct	164	18	10.98	1024	73	7.13	
Nov	142	16	11.27	1584	97	6.12	
Dec	98	11	11.22	1151	58	5.04	
Jan	97	17	17.53	1248	76	6.09	
Feb	139	5	3.60	1952	99	5.07	

**Table 3:** Characteristics of cysts isolated from infected animals.

	Cattle			Buffaloes		
Cysts	Infected animals	Fertile cysts	%	Infected animals	Fertile cysts	%
	80	36	45%	472	128	27.10%
Age (years)						
3–5 year	24	8	33.33%	6	0	0.00%
6 year	19	3	15.79%	49	8	16.33%
7 year	15	7	46.67%	95	26	27.37%
8 years	8	8	100%	162	42	25.93%
9 year	7	4	57.14%	128	46	35.94%
≥10 year	7	6	85.71%	32	6	18.75%
Location						
Thorax muscles	1	1	100%	6	2	33.33%
Heart	1	0	0%	1	0	0%
Kidney	0	0	0%	1	0	0%
Liver	25	11	44%	147	39	26.53%
Lungs	52	23	44.23%	309	84	27.18%
Lungs and liver	1	1	100%	4	3	75%
Skeletal muscles	0	0	0%	1	0	0%
Pancreas	0	0	0%	3	0	0%
Color						
Black	0	0	0	1	0	0%
Green	0	0	0	2		0%
Grey	1	0	0%	9	2	22.22%
Pale yellow	39	15	38.50%	259	44	16.99%
Pinkish yellow	0	0	0%	2	0	0%
Reddish pink	1	0	0%	2	0	0%
Transparent	2	0	0%	14	7	50%
White	32	19	59.38%	175	70	40%
Yellow	5	2	40%	8	5	62.50%

**Table 4:** Interview results including 150 slaughterhouse employees at three urban slaughterhouses in Rawalpindi district.

One Health Factors	Responses					
	Yes		No		Sometimes	
	(*n* *)	%	(*n* *)	%	(*n* *)	%
Proper facilities to dispose of animals’ offal in slaughterhouses	4	2.66	22	14.6	124	82.6
Access of stray dogs to the infected organs	26	17.3	51	34	73	48.6
Veterinary supervision of slaughtered animals	77	51.3	36	24	37	24.6
Health education for butchers	19	12.6	44	29.3	87	58
Stray dogs were fed useless meat (infected)	23	15.3	40	26.6	87	58
Anthelminthic treatment of stray dogs	7	4.6	95	63.3	48	32
Discard infected organs (lungs/liver) at the site of slaughtering	11	7.3	49	32.6	90	60

* *n* is the number of responses out of 150.

## Data Availability

The data presented in this study are available in the manuscript.
